# Supplementary material for: The Ribosome Is the Ultimate Receptor for Trypsin Modulating Oostatic Factor (TMOF)
Source: Biomolecules. 2022 Apr 14;12(4):577. doi: 10.3390/biom12040577 (PMC9028786; doi:10.3390/biom12040577)
Supplement: Supplementary file 1 [file biomolecules-12-00577-s001.zip › biomolecules-1662449-supplementary.pdf]

Supplementary material

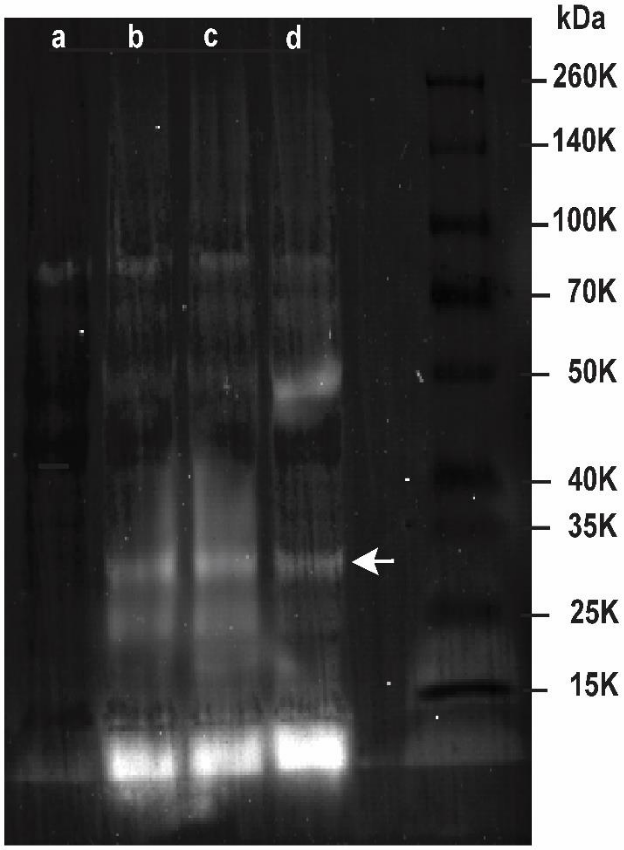

Figure S1. Western blot analysis of *Ae. aegypti* late larval trypsin synthesized in TnT T7 insect cell lysate in the presence of Fluorotech Green $tRNA_{lys}$ and plasmid pFA25A ICE T7 flexi cloned with late larval trypsin lanes (b, c and d). Lane (a) is a control incubation without Fluorotech Green $tRNA_{lys}$. A wide molecular standards (15kDA to 260 kDa) was run on the right. The PVDF membrane was scanned for fluorescence using Biomolecular Imager at 468 nm. White arrow indicates late larval trypsin location at 28.5 kDa. Heavy Fluorescence bands below 15kDa represent Fluorotech Green $tRNA_{lys}$.

A

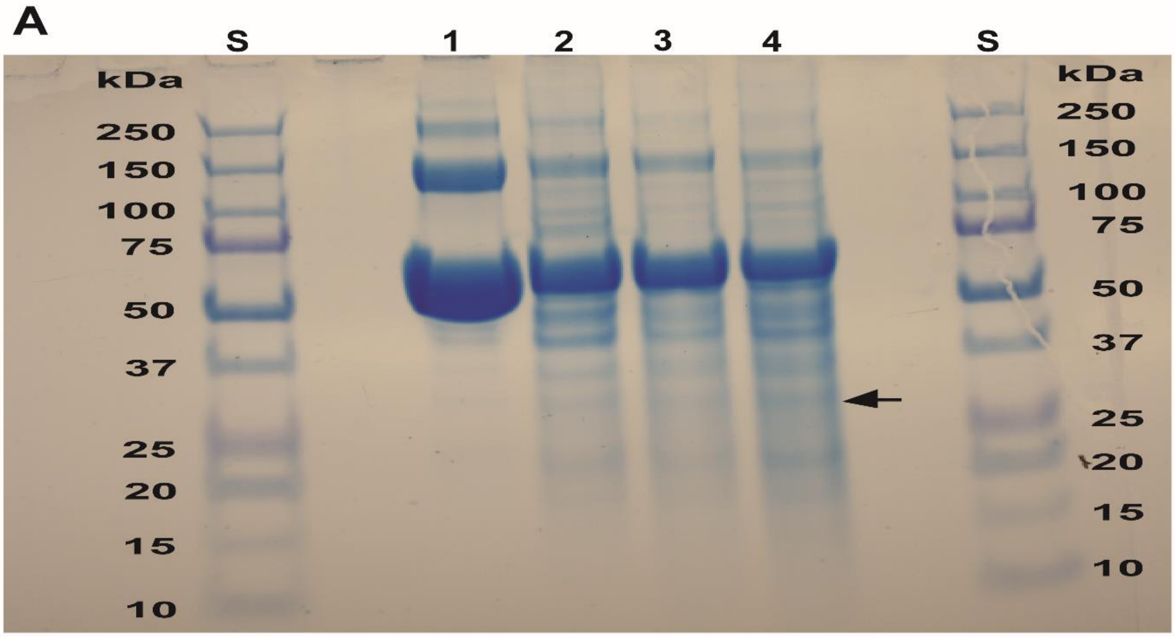

B

```
        10         20         30         40         50         60
MFTSTVVFAS LMALASAFPS LDNGRVVNGQ TATLGQFPFQ VLLKVELSQG RALCGGSLLS

        70         80         90        100        110        120
DQWVLTAGHC ADGAKSFEVT LGAVDFEDTT NDGRVVLTAT EYHRHEKYNP LFATNDVAVV

       130        140        150        160        170        180
KLPTPVEFND RVQPVKLPTG SDTFTDREVV VSGWGLQKNG GDVADKLQYA PLTVISNNEC

       190        200        210        220        230        240
SKAYSPLVIK KTTLCAKGEN KESPCQGDSG GPLVLEGENV QVGVVSFGHA VGCEQGYPGA
```

Figure S2. A. SDS-PAGE of TnT 7T insect cell lysates that were incubated with BSA (lane 1), with cell lysate (lane 2), with empty plasmid pFA25A ICE T7 flexi (lane 3), and plasmid pFA25A ICE-T7 flexi-larval late trypsin (lane 4). Arrow indicates the protein band of *Ae. aegypti* larval late trypsin at 28.5 kDa. **B.** Mass spectrometry analysis of the excised protein band marked with a black arrow (lane 4 above). Seven unique peptide chains (1-25; 26-44; 52-75,76-94;122-131;137-147;159-166) highlighted in yellow were sequenced by MS/MS and the protein identified as late larval trypsin (accession number AAO43403.1).
